# Tumor Marker Heterogeneity in Ovarian Cancer: Clinical Implications of Pretreatment Monitoring of Serum Biomarkers for Recurrence Prediction

**DOI:** 10.14740/wjon2800

**Published:** 2026-07-23

**Authors:** Ahmad Alwazzan, Ahmad Badeghiesh, Majid Almansouri, Dahlia Mirdad, Razan Amjad, Zayd Jastaniah, Huda Altoukhi, Rayyan Hasanain, Abdulkareem Fayoumi, Hatim Almarzouki

**Affiliations:** a Department of Obstetrics and Gynaecology, Faculty of Medicine, King Abdulaziz University, Jeddah, Saudi Arabia; b Department of Obstetrics and Gynaecology, Faculty of Medicine, King Abdulaziz University, Rabigh, Saudi Arabia; c Department of Obstetrics and Gynaecology, Dr. Suliman Fakeeh Hospital, Jeddah, Saudi Arabia; d Department of Clinical Biochemistry, Faculty of Medicine, King Abdulaziz University, Jeddah, Saudi Arabia; e Department of Basic Medical Sciences, College of Medicine, University of Jeddah, Jeddah, Saudi Arabia; f Department of Internal Medicine, Faculty of Medicine, King Abdulaziz University, Rabigh, Saudi Arabia; g Department of Radiology, Faculty of Medicine, King Abdulaziz University, Jeddah, Saudi Arabia

**Keywords:** Ovarian cancer, CA-125, Carcinoembryonic antigen, Lactate dehydrogenase, Pretreatment biomarkers, Prognosis

## Abstract

**Background:**

Ovarian cancer remains a highly lethal gynecological malignancy with heterogeneous clinical outcomes. Reliable pretreatment biomarkers are needed to improve prognostic stratification. This study aimed to evaluate variations in pretreatment serum cancer antigen 125 (CA-125), carcinoembryonic antigen (CEA), and lactate dehydrogenase (LDH) across ovarian cancer subtypes and to determine their ability to predict time to recurrence after treatment.

**Methods:**

This retrospective cohort study included adult patients with histologically confirmed ovarian cancer treated with cytoreductive surgery and adjuvant chemotherapy. Pretreatment serum CA-125, CEA, and LDH levels were measured using standardized immunoassays. Biomarker distributions were analyzed by tumor subtype, and time to recurrence after treatment was assessed using Cox proportional hazards regression analysis and Kaplan–Meier analysis.

**Results:**

The study included 126 ovarian cancer patients (mean age 53.17 ± 15.47 years). Serous carcinoma was most common (46%), with most patients presenting at advanced stage IIIb (44.4%) and 57% having high-grade tumors. Elevated pretreatment CA-125 was observed in 76.7%, while CEA and LDH were less frequent (13.3% and 11.7%). Kaplan–Meier analysis showed that elevated CA-125 was associated with a longer time to recurrence after treatment (log-rank P = 0.0057), whereas CEA (P = 0.853) and LDH (P = 0.562) did not show a statistically significant association. In multivariable Cox proportional hazards regression analysis, LDH was independently associated with a shorter time-to-event (hazard ratio (HR), 1.92; 95% CI, 1.03–3.56; P = 0.039), while CEA showed a modest association (HR, 0.50; 95% CI, 0.25–0.98; P = 0.043). CA-125 did not show a statistically significant association (HR, 0.64; P = 0.072). Suboptimal surgery predicted worse outcomes (HR, 4.79; P < 0.001).

**Conclusions:**

Pretreatment serum CA-125 was not independently associated with time to recurrence after adjustment. Elevated LDH was associated with shorter time-to-event, whereas the prognostic significance of CEA remained uncertain and should be interpreted cautiously. Suboptimal surgical cytoreduction remained strongly associated with worse outcomes. These findings suggest LDH may have prognostic relevance, whereas CA-125 and CEA have limited independent value in this cohort.

## Introduction

Ovarian cancers remain one of the most lethal gynecological malignancies worldwide and is a leading cause of cancer-related mortality among women [1]. This poor prognosis is largely attributable to nonspecific early symptoms, lack of effective screening strategies, and advanced-stage presentation at diagnosis, resulting in high recurrence rates and limited long-term survival [1]. Despite improvements in surgical management and systemic therapy, clinical outcomes remain heterogeneous, highlighting the need for reliable pretreatment biomarkers to improve prognostic stratification and guide therapeutic decisions. Cancer antigen 125 (CA-125) is the most widely used serum biomarker in ovarian cancer with levels exceeding 35 U/mL regarded as abnormal and observed in approximately 90% of ovarian carcinomas [2–5]. CA-125 is also clinically used to monitor response to chemotherapy, detect relapse, and assess disease progression [4, 6]. The prognostic value of pretreatment CA-125 levels, however, remains controversial. Several studies have demonstrated associations between baseline CA-125 concentrations and cancer staging, tumor burden, and histological subtype, with higher positivity rates observed in advanced-stage disease [4, 7]. A large retrospective study reported that higher pretreatment CA-125 levels, particularly values exceeding 500 U/mL in stage III high-grade serous carcinoma, were paradoxically associated with improved survival, emphasizing the complexity of CA-125 interpretation [8]. Several approaches based on CA-125 kinetics have been proposed to assess treatment response, although traditional time-point-based strategies have shown inconsistent predictive value and are limited by biological and analytical variability [9–16].

Beyond CA-125, carcinoembryonic antigen (CEA) has gained interest as a complementary biomarker [17, 18]. Although it is well-established as a prognostic marker in colorectal and other solid tumors, its role in ovarian cancer remains less defined [19, 20]. Elevated pretreatment CEA levels have been associated with mucinous ovarian carcinoma, metastatic disease, and poorer outcomes in selected cohorts [21, 22]. Notably, Lin et al demonstrated that combined pretreatment CA-125 and CEA levels independently predicted both progression-free survival (PFS) and overall survival (OS), with markedly superior outcomes in patients with low levels of both markers [23]. Additionally, the CA-125/CEA ratio reliably differentiates mucinous ovarian carcinoma from other epithelial subtypes [24]. Moreover, lactate dehydrogenase (LDH), a key enzyme in anaerobic glycolysis, has been associated with aggressive tumor behavior and poor prognosis in several malignancies, including ovarian cancer. Its potential role as a pretreatment prognostic biomarker in ovarian cancer warrants further investigation.

The aim of this study is to evaluate the variation of pretreatment serum CA-125, CEA, and LDH levels across ovarian cancer subtypes, including metastatic carcinoma, and to assess their association with time to recurrence after treatment.

## Materials and Methods

This retrospective cohort study was conducted at the Department of Obstetrics and Gynecology of a single tertiary care institution in 2019, following approval by the Institutional Review Board (IRB) committee in King Abdulaziz University, Jeddah, Saudi Arabia (Reference No. 684-19). All procedures were performed in accordance with the Declaration of Helsinki, and written informed consent was obtained from all participants. Adult patients (n = 126), aged ≥ 18 years, with histologically confirmed ovarian cancer who underwent cytoreductive surgery followed by adjuvant chemotherapy were consecutively screened for eligibility. Patients were included if serum levels of CA-125, CEA, and LDH were available at diagnosis and during follow-up, and if a minimum follow-up period of 6 months after completion of chemotherapy was documented. Tumor histological subtype, grade, and stage were classified according to World Health Organization (WHO) criteria and the International Federation of Gynecology and Obstetrics (FIGO) staging system. Postoperatively, patients with primary ovarian cancers received six cycles of adjuvant chemotherapy consisting of carboplatin combined with paclitaxel, while carboplatin monotherapy was reserved for patients unable to tolerate combination therapy. Patients with metastatic tumors were managed according to the treatment protocol for their primary malignancy following multidisciplinary team evaluation. Systemic therapy was individualized based on the site of the primary tumor and contemporary disease-specific treatment guidelines. Follow-up was performed using the same institutional surveillance protocol, including clinical assessment, imaging, and biomarker evaluation when appropriate, to document disease recurrence or progression.

Pretreatment serum CA-125, CEA, and LDH levels were measured using standardized immunoassays on a commercially available platform (Roche Diagnostics, Mannheim, Germany) following manufacturer protocols. Serum biomarker levels were categorized as high or low based on institutional laboratory reference ranges (CA-125 > 35 U/mL, CEA > 5 ng/mL, and LDH > 250 U/L were considered elevated). Treatment response was assessed at completion of chemotherapy, with complete response defined as the absence of detectable disease. Patients were retrospectively followed until the first documented recurrence after treatment. Because reliable information regarding recurrence-free status at the last follow-up or other censoring events was not consistently available, PFS could not be estimated.

### Inclusion and exclusion criteria

Adult women (≥18 years) with histologically confirmed ovarian cancer who underwent primary cytoreductive surgery followed by adjuvant chemotherapy between 2022 and 2024 were eligible for inclusion. Patients were required to have available pretreatment serum CA-125, CEA, and LDH measurements and a documented minimum follow-up of 6 months after chemotherapy completion. Exclusion criteria included incomplete clinical or laboratory data, absence of biomarker follow-up, prior malignancy, neoadjuvant chemotherapy, non-epithelial ovarian tumors, or loss to follow-up before 6 months.

### Statistical analysis

Continuous variables were assessed for their distribution. Normally distributed variables were summarized as mean ± standard deviation (SD), whereas non-normally distributed variables were presented as median and range. Categorical variables were presented as frequencies and percentages. Differences in time to recurrence were evaluated using Kaplan–Meier curves and compared using the log-rank test. Multivariable Cox proportional hazards regression analysis was performed to adjust for potential confounding. The model included pretreatment CA-125, CEA, LDH, age, FIGO stage, tumor grade, histological subtype (serous vs. non-serous), surgical outcome (optimal vs. non-optimal), and treatment regimen (carboplatin plus paclitaxel vs. other/no therapy). These variables were selected based on their established clinical relevance and potential prognostic importance in ovarian cancer. Given the absence of reliable censoring information, the Cox regression analysis was performed primarily to adjust for potential confounding factors, and its findings should be interpreted cautiously. All statistical analyses were conducted using the Statistical Package for the Social Sciences (SPSS) version 26.0 (IBM Corp., Armonk, NY, USA).

## Results

The study cohort consisted of 126 patients with histologically confirmed ovarian cancer who underwent primary surgical resection followed by adjuvant chemotherapy. The mean age at diagnosis was 53.17 ± 15.47 years. Of the 126 patients, 101 (80.2%) had primary ovarian tumors, whereas 25 (19.8%) had metastatic tumors involving ovary. The most common histopathological subtypes of primary ovarian cancers were serous ovarian carcinoma (46.0%) and mucinous ovarian carcinoma (15.1%) (Table 1). According to the FIGO staging system, the majority of patients presented with advanced-stage disease, with stage IIIb (44.4%) being the most frequent stage, and high-grade tumors accounted for 57.1% of cases. Biomarker data (CA-125, CEA, and LDH) were only available for 120 patients due to missing values in six cases.

**Table 1:** Clinical Characteristics of Patients With Ovarian Cancer in Our Study

Category	Frequency (n)	Percent (%)
Age (years), mean ± SD	53.17 ± 15.47	
Histopathological diagnosis		
Dysgerminoma	1	0.8
Endometrioid ovarian carcinoma	17	13.5
Granulosa cell tumor	4	3.2
Immature teratoma	2	1.6
Mucinous ovarian carcinoma	19	15.1
Serous ovarian carcinoma	58	46.0
Metastatic tumor to the ovary	25	19.8
FIGO tumor stage		
I	25	19.8
II	13	10.3
IIb	1	0.8
IIIa	7	5.6
IIIb	56	44.4
IIIc	24	19.0
Tumor grade		
High	72	57.1
Low	17	13.5
Not graded	37	29.4
Baseline CA-125 level^a^		
High (> 35 U/mL)	92	76.7
Low (≤ 35 U/mL)	28	23.3
Baseline CEA level^a^		
High (> 5 ng/mL)	16	13.3
Low (≤ 5 ng/mL)	104	86.7
Baseline LDH level^a^		
High (> 250 U/L)	14	11.7
Low (≤ 250 U/L)	106	88.3
Surgery		
Optimal	40	33.3
Non-optimal	79	66.7
Follow-up duration, median (range)	18.7 months (range:0–111.3)	

^a^CA-125, CEA, and LDH are laboratory-defined biomarkers; data were available for 120 of 126 patients. SD: standard deviation; CA-125: cancer antigen 125; CEA: carcinoembryonic antigen; LDH: lactate dehydrogenase; FIGO: International Federation of Gynecology and Obstetrics.

**Figure 1:**
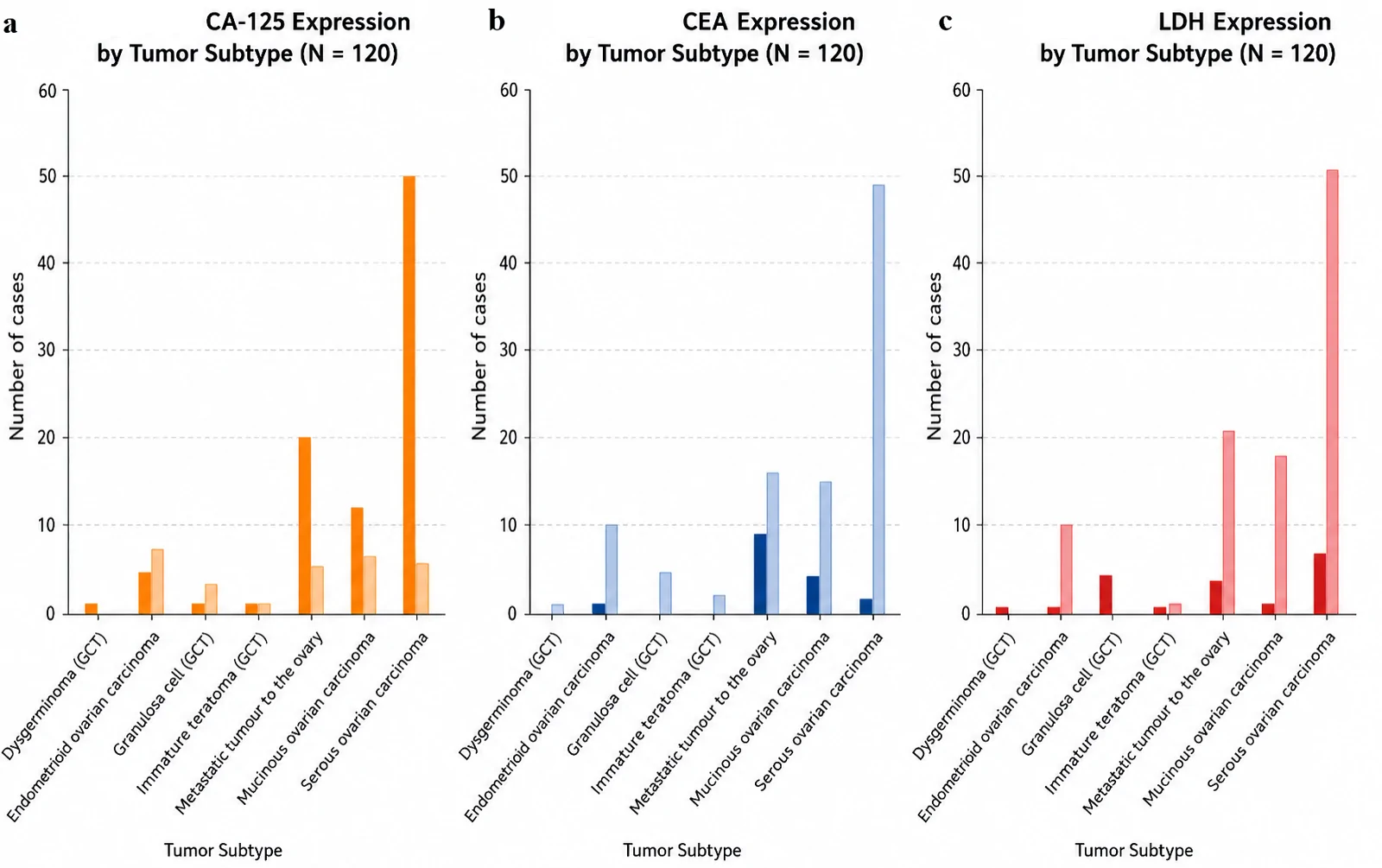
Variation in serum levels of biomarkers CA-125 (a), CEA (b), and LDH (c) across all ovarian tumor subtypes in 120 patients. CA-125: cancer antigen 125; CEA: carcinoembryonic antigen; LDH: lactate dehydrogenase.

At baseline, elevated serum CA-125 levels (> 35 U/mL) were observed in 76.7% of patients. Most cases with elevated pretreatment CA-125 levels were observed in patients with high-grade serous ovarian carcinoma, whereas non-serous subtypes demonstrated lower rates of CA-125 elevation. Elevated CEA and LDH levels were less frequent, occurring in 13.3% and 11.7% of patients, respectively (Table 1). Baseline expression patterns of CA-125, CEA, and LDH varied considerably across ovarian cancer subtypes (Fig. 1). Serous ovarian carcinoma demonstrated the highest number of cases with elevated CA-125, whereas CEA elevation was more frequently observed in mucinous ovarian carcinoma and metastatic tumors to the ovary. In contrast, LDH elevation was uncommon across epithelial subtypes but was more frequently observed in non-epithelial tumors, including germ cell-related neoplasms (Fig. 1). Low-expression patterns predominated for CEA and LDH across most histological categories, whereas CA-125 showed a higher proportion of elevated levels, particularly in serous and metastatic ovarian tumors.

**Figure 2:**
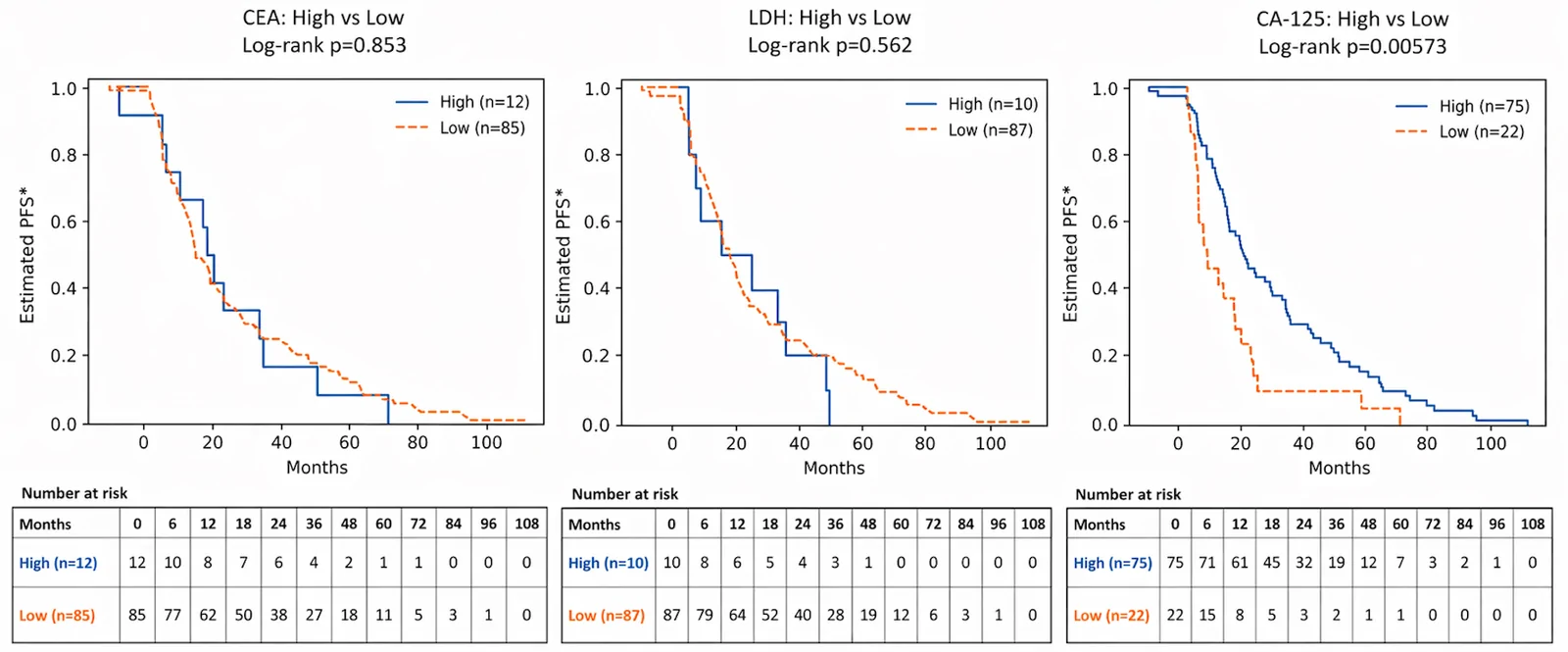
The relationship between pretreatment serum levels of CA-125, CEA, and LDH and prediction of time to recurrence after treatment. PFS: progression-free survival.

Serum CA-125, CEA, and LDH variation across ovarian cancer subtypes and prediction of time to recurrence after treatment were also assessed. Patients with high- versus low-baseline CA-125 levels demonstrated a significant difference in the time to documented recurrence, with elevated CA-125 pretreatment associated with a longer time to recurrence after treatment (log-rank P = 0.0057) (Fig. 2). In contrast, baseline pretreatment CEA (log-rank P = 0.853) and LDH (log-rank P = 0.562) did not show a statistically significant association (Fig. 2). The time to recurrence after treatment was estimated from Kaplan–Meier curves. Patients with elevated CA-125 demonstrated a longer duration compared with those with low CA-125 levels (approximately 23 vs. 13 months). In contrast, no meaningful differences were observed between high and low groups for CEA and LDH.

To account for potential confounding, a fully adjusted Cox proportional hazards model was performed incorporating biomarkers (CA-125, CEA, and LDH) alongside key clinical variables, including age, FIGO stage, tumor grade, histological subtype, surgical outcome, and treatment factors (Table 2). In this model, elevated LDH emerged as an independent predictor of shorter time-to-event (hazard ratio (HR) = 1.92; 95% CI, 1.03–3.56; P = 0.039). Elevated CEA showed a borderline statistical association in the adjusted model (HR = 0.50; 95% CI, 0.25–0.98; P = 0.043); however, this finding should be interpreted with caution given potential residual confounding and the absence of reliable censoring information, which limits the validity of the survival analysis. In contrast, baseline CA-125 was not independently associated with outcome (HR = 0.64; 95% CI, 0.39–1.04; P = 0.072). Among clinical factors, suboptimal surgery remained the strongest predictor of worse outcome (HR = 4.79; 95% CI, 2.82–8.13; P < 0.001). Age, FIGO stage, tumor grade, and histological subtype were not independently associated with survival after adjustment.

**Table 2:** Fully Adjusted Multivariable Cox Proportional Hazards Regression Analysis of Prognostic Factors Associated With Survival in Ovarian Cancer Patients

	Adjusted HR	95% CI	P value
CA-125 high vs low	0.64	0.39–1.04	0.072
CEA high vs low	0.50	0.25–0.98	0.043
LDH high vs low	1.92	1.03–3.56	0.039
Age, per year	1.01	1.00–1.02	0.167
FIGO stage III/IV vs I/II	1.19	0.74–1.89	0.478
High grade vs low/not graded	1.14	0.73–1.78	0.567
Serous vs non-serous histology	0.58	0.37–0.92	0.019
Non-optimal vs optimal surgery	4.79	2.82–8.13	< 0.001
Carboplatin + paclitaxel vs other/no therapy	0.27	0.17–0.44	< 0.001

HR: hazard ration; CI: confidence interval; CA-125: cancer antigen 125; CEA: carcinoembryonic antigen; LDH: lactate dehydrogenase; FIGO: International Federation of Gynecology and Obstetrics.

## Discussion

In this retrospective cohort study, we evaluated the pretreatment serum expression patterns of CA-125, CEA, and LDH across ovarian cancer subtypes and explored their association with clinical outcomes following treatment. The relatively small sample size and uneven distribution of certain histological subtypes, particularly rare tumors, may limit the strength of subgroup analyses and should be considered when interpreting these findings. Our results demonstrated marked heterogeneity in biomarker expression according to histopathological subtype. In unadjusted analysis, elevated pretreatment CA-125 appeared to be associated with a longer time to recurrence after treatment (Fig. 2). However, this observation should be interpreted cautiously. The discrepancy between Kaplan–Meier and Cox regression findings likely reflects differences between unadjusted and adjusted analyses (Table 2). Kaplan–Meier analysis evaluates each variable independently, whereas the multivariable Cox model accounts for key clinical factors, including stage and surgical outcome. After adjustment, CA-125 was no longer independently associated with outcome, suggesting that its apparent effect in the univariate analysis was driven by confounding factors, particularly tumor burden, histological subtype, and other clinicopathological characteristics. The seemingly longer time to recurrence observed among patients with elevated CA-125 in the Kaplan–Meier analysis likely reflects these underlying differences rather than a true protective effect of elevated CA-125. Accordingly, baseline CA-125 appears to be a marker of disease characteristics rather than an independent predictor of recurrence.

In addition to the prognostic analysis, distinct biomarker expression patterns were observed across ovarian cancer subtypes. CA-125 was most frequently elevated in serous ovarian carcinoma, whereas CEA elevation predominated in mucinous carcinoma and metastatic ovarian tumors. In contrast, elevated LDH levels were uncommon in epithelial tumors but occurred more frequently in non-epithelial neoplasms. These findings highlight the biological heterogeneity of ovarian cancer and support the complementary diagnostic value of these biomarkers in different histological subtypes.

Consistent with prior literature, CA-125 remained the most frequently elevated biomarker at diagnosis and demonstrated subtype specificity, with higher expression predominantly observed in serous ovarian carcinoma and metastatic tumors to the ovary [4, 5, 25]. This distribution reflects its origin from coelomic epithelium and supports its established diagnostic role in epithelial ovarian cancer. Similar observations have been reported by Kudoh et al, who demonstrated that CA-125 correlates with tumor burden and varies across histological subtypes [24]. Furthermore, studies evaluating CA-125 kinetics, including KELIM-based analyses, have shown that dynamic changes rather than baseline levels provide more robust prognostic information [26]. In addition to potential residual confounding, the absence of reliable censoring information represents an important methodological limitation, and the Cox regression findings should therefore be interpreted with caution. Multivariable modeling provided adjusted estimates of the associations between clinicopathological variables and survival outcomes within the constraints of the available data. Nevertheless, given the retrospective design, relatively small sample size, and limitations of the survival analysis, these observations should be regarded as preliminary and hypothesis-generating rather than definitive evidence of independent prognostic value.

Pretreatment CEA levels were not significantly associated with outcome in univariate analysis, which is consistent with its known lack of specificity for ovarian epithelial tumors. Elevated CEA was more frequently observed in mucinous carcinoma and metastatic tumors, reflecting gastrointestinal differentiation rather than intrinsic ovarian tumor biology, as reported by Duffy et al [17] and Konishi et al [27]. While Lin et al suggested a combined prognostic role for CEA and CA-125 in selected cohorts enriched for mucinous histology, this effect may not generalize to heterogeneous populations dominated by serous carcinoma [23]. Additionally, prior studies have indicated that the CA-125/CEA ratio is more useful for histological discrimination than for prognostication [28]. Similarly, LDH elevation was uncommon among epithelial ovarian cancers in our cohort and was more frequently associated with non-epithelial tumors, including germ cell neoplasms (Fig. 1). This finding aligns with prior studies demonstrating that LDH and its isoenzyme patterns are particularly informative in dysgerminoma and related tumors [17]. Although LDH reflects tumor metabolism and aggressiveness, its prognostic role in ovarian cancer may only become evident after adjusting for confounding clinical variables.

Overall, these findings suggest that while CA-125 remains a valuable biomarker for disease characterization, its independent prognostic value may be limited. In contrast, LDH and, to a lesser extent, CEA may provide additional prognostic insight when evaluated within a multivariable framework.

### Limitations

Several limitations should be acknowledged. The retrospective, single-center design may introduce selection bias and limit generalizability. The relatively small sample size and heterogeneous distribution of histological subtypes, particularly non-epithelial tumors, may have reduced statistical power for subgroup analyses. Biomarker assessment was restricted to pretreatment levels without evaluation of longitudinal changes for CEA and LDH. Potential confounders, including molecular tumor characteristics and variations in adjuvant therapy, were not fully accounted for. Although most patients received standard platinum-based chemotherapy, treatment variability may have influenced outcomes. Importantly, the absence of reliable censoring information represents an important methodological limitation that may bias hazard ratio estimates and affect the interpretation of time-to-event analyses. Additionally, the proportional hazards assumption was not formally assessed, warranting cautious interpretation of the Cox regression findings. Survival analysis was limited by the absence of censoring data, which may affect the accuracy and interpretability of Kaplan–Meier and Cox regression estimates.

### Conclusions

Pretreatment serum LDH was associated with shorter time-to-event, while CEA showed a modest association that should be interpreted cautiously. Suboptimal surgical cytoreduction also remained a strong independent predictor of worse outcomes. Although findings may have prognostic relevance, the given the relatively small sample size, heterogeneous study population, and retrospective design, these findings should be considered preliminary and require validation in larger, prospective studies before being applied in clinical practice.

## Data Availability

Any inquiries regarding supporting data availability of this study should be directed to the corresponding author.
